# Smoking Induces a Decline in Semen Quality and the Activation of Stress Response Pathways in Sperm

**DOI:** 10.3390/antiox12101828

**Published:** 2023-10-04

**Authors:** Magda Carvalho Henriques, Joana Santiago, António Patrício, Maria Teresa Herdeiro, Susana Loureiro, Margarida Fardilha

**Affiliations:** 1 iBiMED—Institute of Biomedicine, Department of Medical Sciences, University of Aveiro, 3810-193 Aveiro, Portugal; magda.henriques@ua.pt (M.C.H.);; 2 CESAM—Centre for Environmental and Marine Studies, Department of Biology, University of Aveiro, 3810-193 Aveiro, Portugal; 3 Hospital Infante D. Pedro, Centro Hospitalar do Baixo Vouga, EPE, 3810-096 Aveiro, Portugal

**Keywords:** male (in)fertility, semen quality, lifestyle, tobacco, stress response

## Abstract

Male infertility is a prevalent concern affecting couples worldwide. While genetic factors, hormonal imbalances, and reproductive system defects play significant roles, emerging evidence suggests that lifestyle choices also profoundly impact male fertility. This study aimed to explore the effects of several lifestyle factors, including tobacco and alcohol consumption, physical activity, and dietary habits, on semen quality parameters and molecular biomarkers. Thirty healthy male volunteers were recruited in the Urology service at Hospital Infante D. Pedro, Aveiro, Portugal. Participants completed lifestyle questionnaires and provided semen samples, which were analyzed according to the World Health Organization criteria by experienced technicians. We also analyzed the expression levels of antioxidant enzymes and heat-shock response-related proteins to explore the activation of signaling pathways involved in stress response within sperm cells. Our results revealed that tobacco consumption reduced semen volume and total sperm count. Although the changes in the percentage of total motility and normal morphology in the smokers’ group did not reach statistical significance, a slight decrease was observed. Moreover, we identified for the first time a significant association between tobacco consumption and increased levels of heat shock protein 27 (HSP27) and phosphorylated HSP27 (p-HSP27) in sperm cells, indicating the potential detrimental effects of tobacco on the reproductive system. This study highlights that lifestyle factors reduce semen quality, possibly by inducing stress in sperm, raising awareness about the effects of these risk factors among populations at risk of male infertility.

## 1. Introduction

Infertility is a major health problem worldwide, affecting between 8 and 12% of reproductive-aged couples [1]. Recent data from the World Health Organization (WHO) further highlights the widespread impact, indicating that one in six individuals globally has experienced infertility at some point [2]. Remarkably, in approximately 50% of all infertile couples, the male factor is a primary or contributing cause [3]. Various known factors contribute to male infertility, including genetic alterations, anatomical conditions, hormonal deregulations, functional abnormalities of the reproductive system, genital tract infections, immunological and chronic diseases, cancer and related treatments, and sexual disorders that are incompatible with intercourse [4]. However, it is worth noting that about 30–50% of male infertility cases are classified as idiopathic, with no known cause identified. While the exact reasons for idiopathic male infertility are not fully understood, it is believed that various risk factors, including age, psychological stress and quality of life, an inadequate lifestyle, and environmental or occupational exposure to toxicants, may play a role [3,4,5,6,7,8]. Numerous studies have reported that several lifestyle factors, including tobacco smoking [9,10], alcohol consumption [9], and high-fat diets [11], can induce testicular damage, impaired semen parameters, and sperm DNA fragmentation by inducing oxidative stress and inflammation [7,12]. Regarding tobacco smoking, several animal studies have been conducted to understand the effects of cigarette consumption on testes, sperm, and semen parameters. Investigation of the impact of cigarette smoke on the male urogenital system in rat models has revealed its potential to increase oxidative stress levels while causing significant morphological alterations in the testes [13]. In a similar study, authors found that nicotine exposure decreased the sperm count, motility, viability, and normal morphology and induced oxidative stress in the testes and prostate in adolescent male Sprague Dawley rats [14]. Human studies on the effects of tobacco consumption on semen parameters revealed that semen volume, total motility, percentage of morphologically normal spermatozoa, sperm vitality, and sperm membrane integrity were significantly decreased in smokers in comparison with non-smokers [15]. Moreover, several studies have reported that the spermatozoa of smokers have higher levels of DNA fragmentation in comparison with non-smokers [16]. Different mechanisms have been proposed to explain the negative effects of tobacco consumption on semen parameters and sperm DNA integrity, including excessive reactive oxygen species (ROS) production, decreased antioxidant capacity, increased inflammation, and alterations of testicular endocrine function, spermatogenesis, and of the hypothalamic–pituitary axis [17,18]. In a previous study, seminal plasma proteomic profiles were compared between non-smokers and smokers, showing several deregulated proteins, including the inflammation biomarker S100A9 [19]. This suggests a link between smoking, inflammation, and poor semen quality parameters. The inflammation of accessory glands and testes could be related with the observed spermatozoa abnormalities in smokers, such as decreased mitochondrial activity and increased DNA fragmentation [18,19].However, the precise molecular mechanism through which lifestyle impacts sperm quality remains not fully understood.

In cells, protein and cell homeostasis is crucial to maintain the biogenesis, folding, traffic, and degradation [20]. For that, eukaryotic cells possess specialized signaling pathways activated in response to cellular stress conditions, collectively known as the unfolded protein response (UPR) [21,22]. The UPR occurs in the cytosol (cytosolic heat-shock response—HSR), in the endoplasmic reticulum (ER, UPR^ER^), and in the mitochondria (UPR^mt^), often requiring communication with the nucleus. These signaling pathways are crucial for the cells to overcome situations where proteins within the cytosol, ER, and mitochondria are misfolded or accumulate in an unfolded state [23,24]. Nevertheless, the existence of proteins associated with the UPR in spermatozoa remains a subject of debate, primarily due to their apparent transcriptional inactivity and the apparent loss of translational machinery during spermatogenesis, along with the elimination of residual cytoplasm. Indeed, given that sperm cells lack the endoplasmic reticulum (ER) required for protein synthesis, it is likely that any UPR activity would be more pronounced in the cytosol and mitochondria. Santiago et al. [25] reported the presence of UPR-related proteins in human spermatozoa and described that the levels of several proteins from this signaling pathway increased after oxidative stress induction with H_2_O_2_, suggesting the activation of the UPR pathway in spermatozoa in response to stress conditions. However, the question of whether these pathways are active in ejaculated spermatozoa remains uncertain.

Although several lifestyle practices have been associated with increased oxidative stress and declining sperm quality, the exact molecular mechanisms that underlie the impact of lifestyle factors on conventional semen parameters are still not understood [26]. Thus, in the present work, we aimed to investigate the impact of several lifestyle factors on conventional semen parameters and evaluate the activation of stress response pathways in sperm cells under stress conditions, and thus we intended to bridge the existing knowledge gaps concerning the molecular mechanisms that may underlie the impact of unhealthy lifestyle behaviors on male infertility.

## 2. Materials and Methods

### 2.1. Study Design

**Figure 1:**
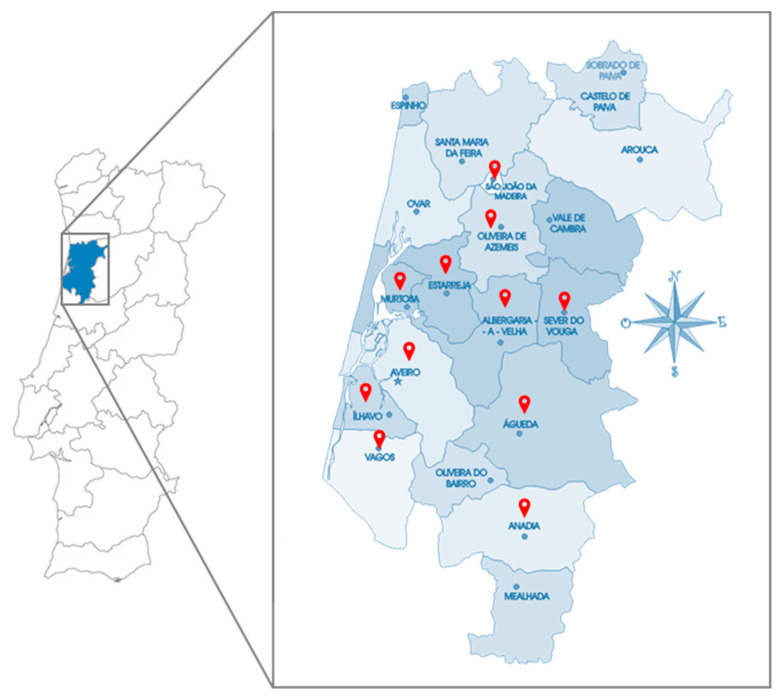
Map highlighting the location of the Aveiro region in Continental Portugal, along with its municipalities. Red symbols (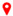) represent the different municipalities of residence of the studied population.

In this cross-sectional study, the effects of several modifiable risk factors (e.g., alcohol and tobacco consumption, exposure to chemicals, etc.) on human semen quality parameters were studied. A total of 30 male volunteers were recruited at the Urology Service at the Hospital Infante D. Pedro E.P.E. (Aveiro, Portugal) between August 2019 and October 2021. Inclusion criteria comprised healthy men of reproductive age and residents in the Aveiro region (Figure 1). The study was approved by the Ethics and Internal Review Board of the Hospital Infante D. Pedro, E.P.E. (Aveiro, Portugal) (Process number: 12-03-2018, date of approval: 2 July 2018) and by The Portuguese Data Protection Authority (Process number: 6617/2018, date of approval: 21 May 2018). Additionally, this study was conducted following the ethical standards of the Helsinki Declaration. No monetary compensation was applied. All data were anonymized. All donors received clear written instructions concerning sample collection and study design and signed informed consent, allowing the use of the samples for scientific purposes. During face-to-face interviews, participants answered a detailed questionnaire regarding sociodemographic (age, body mass index (BMI), etc.), socioeconomic (education, employment status, etc.), diet, and lifestyle, and samples of semen were provided. The questionnaires contained questions regarding sociodemographic information, including age (years); weight (kg); height (cm); municipality of residence; place of residence (rural or urban); time of residence in years; education (primary incomplete, primary complete, high school, or university); employment status (employed, unemployed, or student); and occupational exposure to chemicals (yes or no). The questionnaires also included questions regarding eating and lifestyle habits such as dairy products, vegetables, fruits, fish, shellfish, poultry, meat, and bottled water consumption (never; 1 to 3 times per month; 1 to 3 times per week; 4 to 6 times per week; daily); alcohol and cigarette consumption (yes or no); practice of physical activity (yes or no); vaccination (yes or no); dental filling (yes or no); and medication (yes or no). Finally, information regarding reproductive health was also obtained, namely the duration of sexual abstinence, the number of children, fertility problems (yes or no), and infertility-related conditions (yes or no), and if yes, which condition (for example, varicocele).

### 2.2. Semen Sample Collection, Analysis, and Protein Extraction

Semen samples were obtained from donors by masturbation into a sterile container following a recommended 2–7 days sexual abstinence period. Semen quality analysis was performed according to the WHO guidelines [27] by experienced technicians. Briefly, after complete liquefaction of the semen samples, a macroscopic examination was performed (viscosity, volume, and appearance). Then, a microscopic analysis of sperm motility, concentration, and morphology was performed. All microscopy analyses were performed using a Zeiss Primo Star microscope (Carl Zeiss AG, Oberkochen, Germany). After semen analysis, seminal plasma was separated from sperm cells by centrifugation (600× *g* for 5 min at room temperature (RT)). Then, sperm cells were washed three times in 1× phosphate-buffered saline (PBS) by centrifugation (600× *g* for 5 min at RT). For Western blot, sperm cells were lysed in 1% sodium dodecyl sulphate (SDS) and centrifuged at 16,000× *g* for 20 min at 4 °C. The pellet was stored at −80 °C. The supernatant was used for the subsequent steps (sperm soluble extract). Protein concentration was measured in sperm soluble extract using a bicinchoninic acid assay (Pierce BCA Protein Assay Kit, Thermo Fisher Scientific, Waltham, MA, USA), and final absorbance was measured at 562 nm in a microplate reader (Infinite^®^ 200 PRO series, Tecan Trading AG, Männedorf, Switzerland).

### 2.3. Slot Blot Analysis

Sperm protein lysates (5 µg) were blotted under vacuum into a nitrocellulose membrane, 0.45 μm pore size (GE Healthcare, Chicago, IL, USA), inside the slot blot device (BioRad Portugal, Sintra, Portugal). The membranes were blocked with 5% (*w*/*v*) Bovine Serum Albumin (BSA) in tris-buffered saline containing 0.1% Tween 20 (TBS-T) or 5% (*w*/*v*) non-fat milk in TBS-T for 1 h at RT, incubated for 1 h at RT with primary antibodies (Table 1), and then for 1 h at RT with appropriate secondary antibodies. The infrared IRDye^®^680RD anti-rabbit (926-68071) and IRDye^®^800CW anti-mouse (926-32210) secondary antibodies (1:15,000) were obtained from LI-COR Biosciences (Lincon, NE, USA). Membranes were scanned using the Odyssey Infrared Imaging System (LI-COR^®^ Biosciences, Lincon, NE, USA). Results were normalized to Ponceau staining used before antibody probing. Phospho-proteins were normalized against the total level of the target protein.

### 2.4. Statistical Analysis

A total of 30 healthy male volunteers were recruited for this study. The dataset size was considered reasonable, as it aligns with the principles of the central limit theorem, allowing us to reasonably approximate the distribution of variables to a normal distribution. Descriptive statistical analysis of all data was conducted to characterize the sample and detect possible extreme outliers and measurement errors using IBM SPSS statistics software version 27.0 for Windows. The effect of lifestyle behavior on the semen quality parameters was investigated using redundancy analysis (RDA). Forward selection was used in RDA to select the subset of variables that provided the best explanatory model, and variables with *p* < 0.05 were included in the constrained ordination model. These analyses were performed using R Statistical Software version 4.2.2 in RStudio version 2022.12.0. According to the RDA results, the study participants were divided into two groups: non-smokers and smokers, who were similar in terms of age and BMI. The normality of the data was assessed using the Shapiro–Wilk test. The statistical significance of the effects of tobacco consumption on conventional semen parameters and protein levels was evaluated using tests of the equality of means for paired samples: Student’s *t*-test (parametric test) and Mann–Whitney U test (non-parametric test). A *p*-value <0.05 was set as the level of statistical significance, and these statistical analyses were also performed using IBM SPSS statistics software version 27.0 for Windows.

## 3. Results

A total of 30 healthy male volunteers with a mean age of 32.5 ± 7.4 years were included in this study. A descriptive analysis of the sociodemographic and socioeconomic data, exposure to chemicals at work, lifestyle factors, and conventional semen quality parameters is presented in Table 2. Regular smoking, alcohol consumption, and physical activity habits were reported by 47%, 53%, and 57% of the participants, respectively. Regarding semen quality parameters, the median sperm concentration, total count, total motility (P and NP), and normal morphology were 47 million/mL, 133 million, 59%, and 6%, respectively. According to the WHO reference values, 37% (*n* = 11), 40% (*n* = 12), and 27% (*n* = 8) of participants had sperm concentration, sperm count, and sperm progressive motility below the reference values, respectively. In total, 14 participants (47%) had at least one semen quality parameter (volume, concentration, total count, total motility, and/or morphology) below the reference values.

Concerning the dietary habits of the male volunteers (Table 3), coffee was the most frequently consumed beverage, with 77% of participants drinking it daily, followed by bottled water (64%). The consumption of fresh vegetables, fruit, or fruit juices varied, with 90% of participants consuming them between one and three times per week or daily. Poultry, eggs, and fresh or frozen fish were consumed by more than 60% of participants, typically one to three times per week.

### 3.1. Impact of Lifestyle on the Conventional Semen Quality Parameters

**Figure 2:**
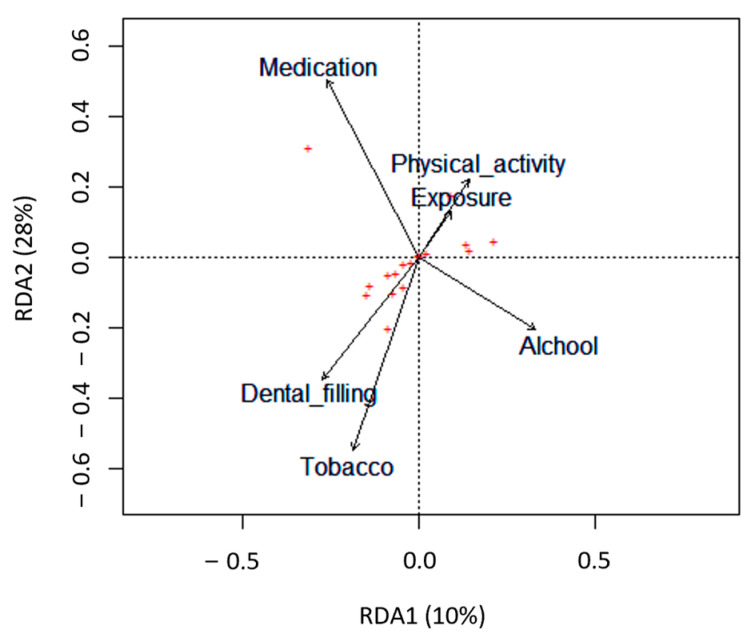
Biplots based on redundancy analysis (RDA) represent the correlation between the semen quality parameters (red crosses, **+**) and the lifestyle factors analyzed (tobacco, alcohol, dental filling, exposure to chemicals at work, physical activity, and medication). Lifestyle factors are represented by arrows that indicate the direction in which the variables are increasing.

The impact of several lifestyle factors, including age, BMI, alcohol and tobacco consumption, physical activity, and dietary habits, on semen parameters was evaluated using a redundancy analysis (RDA) (Figure 2).

**Figure 3:**
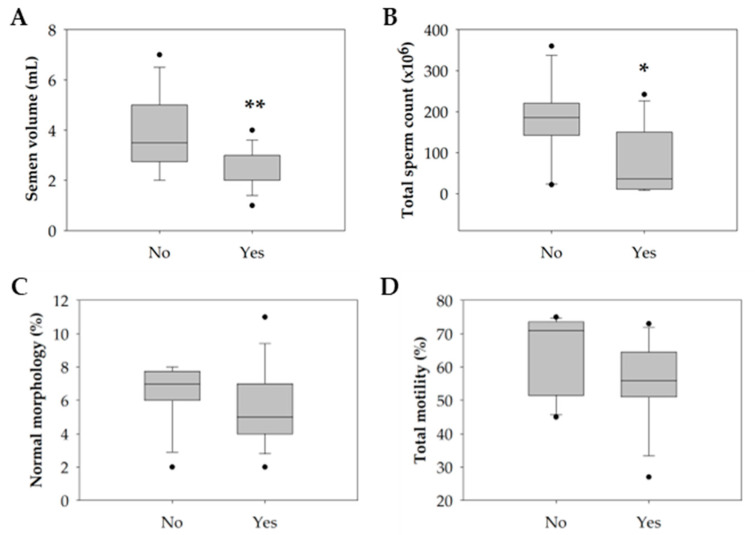
Boxplot illustrating the distribution of the (**A**) semen volume (mL). (**B**) Total sperm count (×10^6^). (**C**) Percentage of sperm with normal morphology (%). (**D**) Percentage of sperm total motility (%) among non-smokers (*n* = 16) and smokers (*n* = 14). The horizontal line represents the median, the box edges indicate the 25th and 75th percentiles, the whiskers extend to encompass the smallest and highest values within a range of 1.5 times the length of the box, and the dots (●) indicate outliers which are values outside 1.5 the interquartile range. Asterisks (*) represent significant differences compared to non-smokers (* *p* < 0.05; ** *p* < 0.01).

Only tobacco consumption significantly negatively impacted semen quality parameters (*p* = 0.047). Particularly in the smokers’ group, there was a significant decrease in semen volume (*p* = 0.004) and the total count of spermatozoa in the ejaculate (*p* = 0.044) compared with the non-smokers’ group (Table 4 and Figure 3A,B). Concerning the percentage of spermatozoa with normal morphology and total motile spermatozoa, although not statistically significant, the smokers’ group revealed a slight reduction compared with the non-smokers’ group (Table 4 and Figure 3C,D).

### 3.2. Smoking Increases the Phosphorylation of HSP27

**Figure 4:**
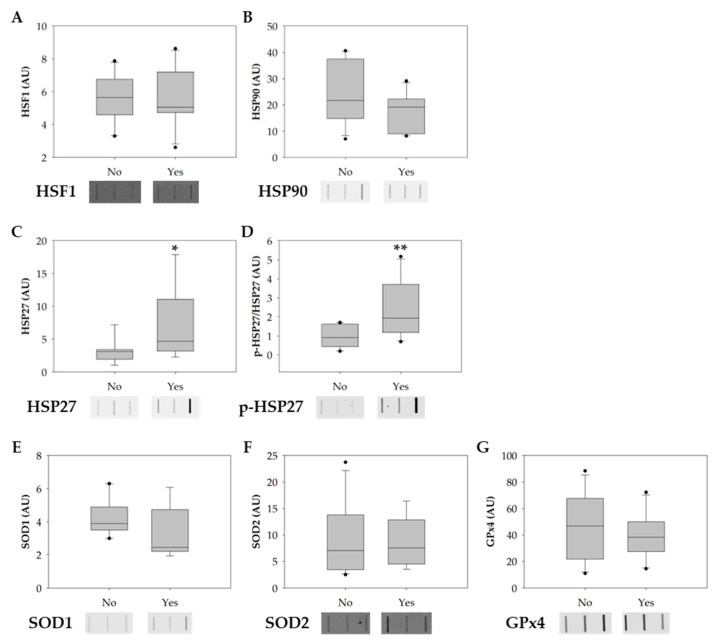
Boxplots showing the effect of tobacco consumption on the protein levels of (**A**) HSF1, (**B**) HSP90, (**C**) HSP27, (**D**) p-HSP27, (**E**) SOD1, (**F**) SOD2, and (**G**) GPx4. Ponceau S. was used as the protein-loading control for data normalization. Phosphorylation-specific signals were normalized against the total level of the protein. The horizontal line displays the median, the box edges show the 25th and 75th percentiles, and the whiskers show the smallest and highest value within 1.5 box lengths from the box (*n* = 11 for non-smokers; *n* = 10 for smokers). The horizontal line represents the median, the box edges indicate the 25th and 75th percentiles, the whiskers extend to encompass the smallest and highest values within a range of 1.5 times the length of the box, and the dots (●) indicate outliers which are values outside 1.5 the interquartile range. Asterisks (*) represent significant differences compared to non-smokers (* *p* < 0.05; ** *p* < 0.01).

The protein levels of heat-shock factor 1 (HSF1), heat- shock protein 90 (HSP90), heat shock protein 27 (HSP27), phosphorylated HSP27 (p-HSP27), copper-zinc superoxide dismutase also known as superoxide dismutase 1 (SOD1), mitochondria SOD (SOD2), and phospholipid hydroperoxide glutathione peroxidase (GPx4) were evaluated among smokers and non-smoker groups to explore possible molecular alterations in sperm induced by tobacco consumption. In the smokers’ group, there was a significant increase in the levels of HSP27 and p-HSP27 (Figure 4A,B). No significant alterations were observed in the levels of HSF1, HSP90, SOD1, SOD2, and GPx4 between groups (Figure 4D–F).

## 4. Discussion

Over the past few decades, a decline in semen quality parameters has been reported [28,29]. Increasing evidence suggests that lifestyle-related factors, such as tobacco, alcohol, and recreational drug consumption, obesity, advanced paternal age, physical activity, and dietary habits, play a significant role in this decline by inducing testicular damage, impaired semen parameters, and sperm DNA fragmentation [7,9,10,11,12,30]. In the current study, we investigated the impact of several lifestyle factors, such as tobacco and alcohol consumption, the practice of physical activity, and dietary habits, on semen quality parameters. According to the RDA performed, tobacco consumption was found to be negatively correlated with conventional semen quality parameters, particularly causing a significant decrease in semen volume and total sperm count. Consistent with our findings, several meta-analyses and literature reviews have reported that tobacco consumption is associated with reduced semen volume, sperm concentration, total sperm count, total motility, and percentage of morphological normal spermatozoa, both among healthy men and infertile men [31,32,33]. The decrease in total sperm count observed among the smokers’ group, can be attributed to the decrease in semen volume, as the total sperm count is dependent on it. Several studies have also reported a decrease in semen volume in smokers when compared with non-smokers [34,35,36]. This reduction in semen volume observed among smokers may be a result of the impact of nicotine, which is present in cigarettes, on accessory glands, such as the seminal vesicle, prostate, and urethral glands [37]. Previous studies have shown that smokers have significantly lower ultrasound-derived seminal vesicle volume when compared with non-smokers [38]. Since accessory glands play a crucial role in controlling semen volume through their secretions, the effects of nicotine on these glands could contribute to the observed decrease in semen volume among smokers. Additionally, although without statistical significance, we also found a slight reduction in the percentage of sperm with normal morphology and total motility in the sperm samples from the smokers’ group compared to non-smokers. Several studies have demonstrated that tobacco consumption has been associated with impaired sperm maturation and sperm function and negatively impacts the semen parameters (reviewed by [32]). These effects include a decrease in sperm count, concentration, motility and viability; an increased number of spermatozoa with morphological defects; and a reduced capacity of spermatozoa to undergo capacitation and acrosome reactions (reviewed by [12]).

Despite the available knowledge on the adverse effect of tobacco on sperm parameters, the molecular mechanisms underlying sperm’s response to tobacco exposure are not fully understood. Several studies suggest that the detrimental effects of tobacco consumption on semen parameters are partly attributed to the increased generation of reactive oxygen species (ROS) in the testes, induced by the contents of tobacco (e.g., nicotine) (as reviewed by [12,30]). The abnormal accumulation of ROS in the testes has adverse effects on both spermatogenesis and steroidogenesis, resulting in reduced sperm production and a subsequent decline in overall sperm count. Furthermore, spermatozoa are particularly vulnerable to ROS due to their limited antioxidant defense mechanisms [39]. Consequently, oxidative stress significantly impairs sperm function by compromising its viability and motility, inducing DNA fragmentation, and promoting membrane lipid peroxidation [40]. In this study, the expression levels of proteins involved in sperm stress response, such as antioxidant enzymes (SOD1, SOD2, and GPx4) and heat-shock response (HSR)-related proteins (HSF1, HSP90, HSP27, and p-HSP27), were evaluated to elucidate the impact of consumption of tobacco in spermatozoa. No significant alterations in the expression levels of the antioxidant enzymes SOD1, SOD2, and GPx4 were found. However, it is essential to consider, when interpreting these results, that even though no significant changes in the expression levels of SOD1, SOD2, and GPX4 were observed in our work, their activity may still be altered. In line with this, a previous study showed that the sperm enzymatic activity of GPx4 was significantly decreased in the sperm of male smokers when compared with non-smokers [41]. A similar pattern was observed in the mRNA expression of GPx4 in male smokers when compared with non-smokers. In another study, the authors found that smoking was significantly associated with decreased seminal SOD activity and decreased SOD2 mRNA expression in sperm cells [42]. Several other studies showed that tobacco consumption induced a decrease in the activity of SOD, CAT, and GST in seminal plasma [42,43,44,45]. Together, the results presented in these studies suggest that tobacco consumption has an adverse effect on the antioxidant enzymes activity, leading to an increase in the accumulation of ROS and consequently oxidative stress in sperm [41,42].

**Figure 5:**
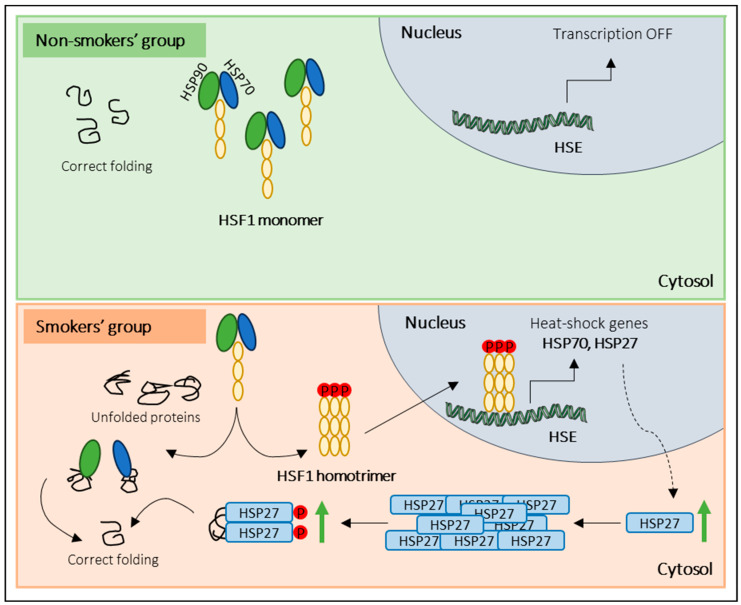
Overview of the proposed sperm response to tobacco exposure. In the non-smokers’ group (non-stress conditions), monomeric HSF1 exists in an inactive form associated with molecular chaperones (HSP90 and HSP70). In response to tobacco consumption (stress conditions), HSF1 dissociates from the molecular chaperones, is phosphorylated, and forms homotrimers that are translocated into the nucleus. Inside the nucleus, HSF1 binds to heat-shock elements (HSE) and initiates the transcription of heat-shock genes (e.g., HSP27). Once transcribed, HSP27 forms oligomeric structures and, after phosphorylation (P), reorganizes into small dimers that bind misfolded proteins.

Regarding the activation of other stress response pathways, our results showed increased levels of total and phosphorylated HSP27 in the smokers’ group. Cumulative evidence support the notion that under stress conditions, the heat-shock response (HSR) pathway is activated in somatic cells to protect the cytosolic protein-folding environment [25,46]. Briefly, under non-stress conditions, HSF1 exists in an inactive monomeric form associated with the cytosolic chaperones, namely, heat-shock 70 kDa protein I (HSP70/HSPAI) and heat shock protein 90 (HSP90). Indeed, under stress conditions, HSF1 dissociates from the chaperones, is phosphorylated, and assembles into homotrimers that are subsequently translocated into the nucleus. Inside the nucleus, HSF1 initiates the transcription of HSP genes, including HSP70, HSP90, and small HSPs, namely HSP27 [47]. Once transcribed, HSP27 forms large oligomeric structures and reorganizes after phosphorylation (P) into small dimers that bind misfolded proteins [46,48]. Several studies have proved that HSP27 is essential in cell protection against several stressors, such as high temperatures, oxidants, heavy metals, and bacterial and viral infections [25,46,49]. Although, in the current study, no significant alterations in the levels of HSF1 and HSP90 were found, the increased expression levels of total HSP27 and p-HSP27 in the smokers’ group let us hypothesize that tobacco consumption induces the activation of HSR in sperm cells in response to stress (Figure 5). However, to confirm the activation of the HSR pathway, we propose to evaluate the expression levels of the phosphorylated form of HSF1 instead of the total protein, as we did in this study. The evaluation of phosphorylated HSF1 is crucial because it is expected that this form will be the predominant and most relevant form under stress conditions. Additionally, our results challenge the conventional belief of transcription and translation inactivity in sperm cells, raising questions about the observed increase in protein levels post-cellular stress. While mature sperm cells are traditionally seen as translationally inert due to chromatin packaging by protamines, a previous study [50] demonstrates the incorporation of labelled amino acids into polypeptides during sperm capacitation. Inhibiting protein translation has profound effects on sperm motility, capacitation, and in vitro fertilization rates, suggesting translation’s significance in these processes [50]. These findings were confirmed by Zhu et al. [51], who noted reduced mitochondrial protein levels and slower sperm motility when mitochondrial protein synthesis was inhibited. These discoveries suggest that sperm may synthesize new proteins in response to cellular stress, potentially correcting misfolded proteins to prevent damage. Future research should explore mRNA levels in sperm after oxidative stress and assess whether translation inhibitors can mitigate the observed protein level increases under stress conditions. This inquiry illuminates sperm biology’s response to cellular stress, offering promising insights.

To the best of our knowledge, there are no apparent studies concerning the potential activation of HSR in sperm cells among male smokers. Nevertheless, our findings enhanced our understanding of the underlying pathophysiological mechanisms responsible for the decline in semen quality parameters induced by specific lifestyle behaviors. In particular, our results suggest a correlation between tobacco consumption and a decrease in semen quality parameters, namely a reduction in semen volume and total sperm count. Furthermore, it appears that tobacco consumption may trigger the activation of signaling pathways as a response to the stressful conditions induced by such consumption. However, it is crucial to consider that this study has several limitations. Firstly, the sample size of this study is relatively small. A larger and more diverse sample would provide a more robust representation of the population and increase the generalizability of the findings. Secondly, this study employs a cross-sectional design, which limits our ability to establish causality and only allows for the observation of associations at a single point in time. Third, the reliance on self-reported data for variables such as tobacco consumption and other lifestyle behaviors introduces the potential for recall bias and social desirability bias, which could potentially compromise the accuracy of the reported behaviors. To gain a comprehensive understanding of the impact of unhealthy lifestyle behaviors on conventional semen parameters and their role in the decline of male fertility, further studies with a larger sample size are needed. These studies should also analyze the integrity of DNA within sperm cells and explore whether semen quality parameters can recover following the cessation of these detrimental behaviors or if there are persistent long-term impacts. By conducting such research, we can better comprehend the implications of unhealthy lifestyle choices on male fertility decline and develop appropriate interventions or preventive measures.

## 5. Conclusions

In conclusion, our study supports the idea that tobacco consumption negatively affects conventional semen quality parameters, specifically leading to a reduced semen volume and total sperm count. We also showed, for the first time, that tobacco consumption significantly increases the expression levels of proteins involved in the HSR, specifically total and phosphorylated HSP27. These findings provide a first insight into the possible activation of HSR in human spermatozoa in response to the harmful effects of tobacco consumption on sperm cells. Thus, our research suggests that the decline in sperm quality, which is often observed in individuals with unhealthy lifestyle habits, in this case, tobacco consumption, can potentially be explained by the increase in protein aggregation in sperm cells and consequent activation of stress response mechanisms. Understanding the link between poor lifestyle choices and reduced sperm quality clarifies the importance of adopting healthier behaviors. Furthermore, continued research in this area could increase our understanding of the adverse effects of tobacco on male reproductive health and potentially unveil new options for interventions and preventive strategies.

## Figures and Tables

**Table 1:** Primary antibodies used for Immunoblot.

Antibody	Host	Dilution	Supplier	Cat. No
SOD1	Mouse	1:1000	Millipore	MABC684
SOD2	Rabbit	1:1000	Abcam	Ab13533
HSP27	Mouse	1:1000	Santa Cruz Biotechnology	sc-13132
p-HSP27 (Ser82)	Mouse	1:1000	Santa Cruz Biotechnology	sc-166693
GPx4	Rabbit	1:1000	Millipore	ABC269
HSF1	Mouse	1:500	Santa Cruz Biotechnology	sc-17757
HSP90	Mouse	1:2000	ProteinTech	13171-1-AP

**Table 2:** Descriptive analyses of questionaries’ data and semen quality parameters of male volunteers. Data are presented as mean ± standard error (SE) or percentage.

Parameter		N
Age (years)	32.5 ± 7.4	30
BMI (kg/m^2^)	24.3 ± 4.0	30
Education		
Primary	10	3
High School	63	19
University	27	8
Employment Situation		
Employed	90	27
Unemployed	3	1
Student	7	2
Exposure to chemicals at work		
No	47	14
Yes	53	16
Smoking status		
No	53	16
Yes	47	14
Alcohol consumption		
No	47	14
Yes	53	16
Physical activity		
No	43	13
Yes	57	17
Semen parameters		
Sexual abstinence (days)	4 ± 1	29
Volume (mL)	3.1 ± 1.4	30
Concentration (×10^6^/mL)	47 ± 36	28
Total spermatozoa number (×10^6^)	133 ± 110	28
Total motility (Progressive and non-progressive, %)	59 ± 2	29
Progressive motility (%)	42 ± 15	29
Non-progressive motility (%)	17 ± 9	29
Immotile (%)	41 ± 13	29
Normal morphology (%)	6 ± 2	28
Head defects (%)	86 ± 5	28
Midpiece defects (%)	50 ± 10	28
Tail defects (%)	26 ± 7	28
Teratozoospermia index	1.72 ± 0.15	28

**Table 3:** Frequency of consumption of certain food groups by the male participants. Data are presented as percentages.

Consumed Items	Never	1 to 3 Times per Month	1 to 3 Times per Week	4 to 6 Times per Week	Daily
Dairy products	3	17	23	23	33
Coffee	3	3	10	3	77
Tea	27	43	20	3	7
Eggs	3	27	63	7	0
Fresh vegetables	3	0	40	43	13
Canned vegetables	27	30	30	13	0
Fresh fruit or fruit juices	0	10	23	27	40
Canned fruit	53	43	3	0	0
Refrigerants	23	30	17	7	23
Organic food	17	30	20	23	10
Fast-food	10	70	17	0	0
Poultry	0	0	63	33	3
Meat	0	23	50	23	3
Fresh or frozen fish	0	20	63	17	0
Canned fish	40	37	23	0	0
Shellfish	17	63	20	0	0
Grilled food	3	33	47	13	3
Smoked food	3	53	30	7	7
Bottled water	10	3	3	7	77

**Table 4:** Influence of tobacco consumption on semen parameters. The results are expressed as the mean ± standard error. * *p* < 0.05; ** *p* < 0.01.

Parameter	Non-Smokers (N = 16)	Smokers (N = 14)	*p*-Value
Age (years)	31.7 ± 7.9	33.4 ± 6.7	0.545
BMI (kg/m^2^)	25.2 ± 4.9	23.2 ± 2.5	0.184
Semen quality parameters			
Volume (mL)	3.7 ± 1.5	2.3 ± 0.9	0.004 **
Concentration (×10^6^/mL)	54.5 ± 34.4	38.5 ± 36.1	0.242
Total spermatozoa count (×10^6^)	181.9 ± 113.0	84.3 ± 85.1	0.044 *
Total motility (Progressive and non-progressive, %)	62.6 ± 13.9	55.8 ± 11.6	0.093
Progressive motility (%)	46.2 ± 14.4	38.3 ± 15.0	0.159
Non-progressive motility (%)	17.1 ± 7.7	17.5 ± 10.6	0.813
Immotile (%)	37.4 ± 13.9	44.2 ± 11.6	0.093
Normal morphology (%)	6.4 ± 1.6	5.4 ± 2.2	0.069
Head defects (%)	85.2 ± 5.5	86.3 ± 4.8	0.210
Midpiece defects (%)	50.7 ± 9.8	50.1 ± 10.2	0.881
Tail defects (%)	25.5 ± 7.6	25.6 ± 6.0	0.913
Teratozoospermia index	1.72 ± 0.17	1.72 ± 0.13	0.891

## Data Availability

The data presented in this study are available in the present article.
